# 2320. Novel checkpoint modifier lowers T cell activation threshold and enhances and broadens vaccine-induced responses to chronic viral infections

**DOI:** 10.1093/ofid/ofac492.151

**Published:** 2022-12-15

**Authors:** Mohadeseh Hasanpourghadi, Andrew Luber, Sue Currie, Colin Magowan, Xiangyang Zhou, Hildegund C J Ertl

**Affiliations:** The Wistar Institute, Philadelphia, Pennsylvania; Virion Therapeutics, Newark, Delaware; Virion Therapeutics, Newark, Delaware; Virion Therapeutics, Newark, Delaware; The Wistar Institute, Philadelphia, Pennsylvania; The Wistar Institute, Philadelphia, Pennsylvania

## Abstract

**Background:**

Vaccines aim to induce immune responses that prevent disease. They may also clear chronic infections or reduce tumor progression. Vaccine adjuvants augment immune responses, in general, by providing stimulatory signals. Our focus has been on a different type of adjuvant that enhances vaccine-induced T cell responses by modulating the herpes virus entry mediator (HVEM) pathway. The B and T cell attenuator (BTLA) is expressed on naïve T cells and, upon binding to HVEM on antigen presenting cells, dampens signaling through the T cell receptor. HVEM binds with a different domain to the co-stimulator LIGHT. Within a trimolar BTLA-LIGHT/HVEM complex, inhibition prevails. Herpes simplex virus (HSV-1) glycoprotein D (gD) attaches to the BTLA binding site of HVEM and as it has higher binding affinity outcompetes BTLA binding and allows for co-stimulation through LIGHT. This results in enhanced signaling through the T cell receptor and thereby augments and broadens CD8+ T cell responses as we showed with chimpanzee adenovirus (AdC) vector vaccines for several viral antigens.

**Methods:**

Immunogenicity in rodents was evaluated following one or two immunizations with AdC vectors expressing antigens of HIV (gag), HPV-16 (E7/6/5), HBV (core & pol), influenza virus (nucleoprotein) and SARS-CoV2 (nucleoprotein), with or without gD. Vaccine-induced CD8^+^ T cell responses, including their magnitude, functions, duration, and breadth were characterized. Vaccine efficacy was also evaluated.

**Results:**

Vaccination with gD-antigen fusion proteins increased CD8^+^ T cell frequencies to all of the antigens tested (Fig) and improved efficacy. Addition of gD increased stimulation of CD8^+^ T cells to subdominant epitopes and thereby enhanced breadth of responses.

**Conclusion:**

Checkpoint modification of the HVEM pathway with a gD-antigen fusion protein produces potent, prolonged, and broad responses of CD8^+^ T cells to immunodominant and subdominant epitopes. The latter is especially important for chronic viral infections, where, due to exhaustion of T cells to dominant epitopes therapeutic efficacy of vaccines may rely on expansion of T cells to subdominant epitopes. Clinical studies to evaluate therapeutic vaccination for chronic HBV are planned.

**Disclosures:**

**All Authors**: No reported disclosures.

**Figure.**
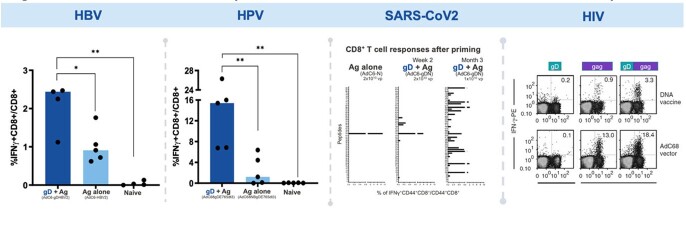
CD8+ T cell Responses, Post Vaccination, Across Multiple Chronic Viral Diseases

